# Paving Luteolin Therapeutic Potentialities and Agro-Food-Pharma Applications: Emphasis on *In Vivo* Pharmacological Effects and Bioavailability Traits

**DOI:** 10.1155/2021/1987588

**Published:** 2021-09-20

**Authors:** Yasaman Taheri, Javad Sharifi-Rad, Gizem Antika, Yakup Berkay Yılmaz, Tugba Boyunegmez Tumer, Sawsan Abuhamdah, Subhash Chandra, Sarla Saklani, Ceyda Sibel Kılıç, Simona Sestito, Sevgi Durna Daştan, Manoj Kumar, Mohammed M. Alshehri, Simona Rapposelli, Natália Cruz-Martins, William C. Cho

**Affiliations:** ^1^Phytochemistry Research Center, Shahid Beheshti University of Medical Sciences, Tehran, Iran; ^2^Graduate Program of Molecular Biology and Genetics, Institute of Natural and Applied Sciences, Canakkale Onsekiz Mart University, Canakkale, 17020, Turkey; ^3^Graduate Program of Biomolecular Sciences, Institute of Natural and Applied Sciences, Canakkale Onsekiz Mart University, Canakkale, 17020, Turkey; ^4^Department of Molecular Biology and Genetics, Faculty of Arts and Science, Canakkale Onsekiz Mart University, Canakkale, 17020, Turkey; ^5^College of Pharmacy, Al-Ain University, Abu Dhabi, UAE; ^6^Department of Biopharmaceutics and Clinical Pharmacy, School of Pharmacy, The University of Jordan, Amman, Jordan; ^7^Department of Pharmaceutical Chemistry, School of Sciences, H. N. B. Garhwal (A Central University), Srinagar Garhwal, Uttarakhand, India; ^8^Department of Pharmaceutical Botany, Faculty of Pharmacy, Ankara University, Ankara 06100, Turkey; ^9^Department of Chemistry and Pharmacy, University of Sassari, via Muroni 23a, 07100 Sassari (SS), Italy; ^10^Department of Biology, Faculty of Science, Sivas Cumhuriyet University, 58140, Sivas, Turkey; ^11^Beekeeping Development Application and Research Center, Sivas Cumhuriyet University, 58140, Sivas, Turkey; ^12^Chemical and Biochemical Processing Division, ICAR-Central Institute for Research on Cotton Technology, Mumbai-400019, India; ^13^Pharmaceutical Care Department, Ministry of National Guard-Health Affairs, Riyadh, Saudi Arabia; ^14^Department of Pharmacy, University of Pisa, via Bonanno 6, 56126 Pisa (PI), Italy; ^15^Faculty of Medicine, University of Porto, Alameda Prof. Hernani Monteiro, 4200-319, Porto, Portugal; ^16^Institute for Research and Innovation in Health (i3S), University of Porto, 4200-135 Porto, Portugal; ^17^Institute of Research and Advanced Training in Health Sciences and Technologies (CESPU), Rua Central de Gandra, 1317, 4585-116 Gandra PRD, Portugal; ^18^Department of Clinical Oncology, Queen Elizabeth Hospital, Kowloon, Hong Kong

## Abstract

Luteolin is a naturally occurring secondary metabolite belonging to the class of flavones. As many other natural flavonoids, it is often found in combination with glycosides in many fruits, vegetables, and plants, contributing to their biological and pharmacological value. Many preclinical studies report that luteolin present excellent antioxidant, anticancer, antimicrobial, neuroprotective, cardioprotective, antiviral, and anti-inflammatory effects, and as a consequence, various clinical trials have been designed to investigate the therapeutic potential of luteolin in humans. However, luteolin has a very limited bioavailability, which consequently affects its biological properties and efficacy. Several drug delivery strategies have been developed to raise its bioavailability, with nanoformulations and lipid carriers, such as liposomes, being the most intensively explored. Pharmacological potential of luteolin in various disorders has also been underlined, but to some of them, the exact mechanism is still poorly understood. Given the great potential of this natural antioxidant in health, this review is aimed at providing an extensive overview on the *in vivo* pharmacological action of luteolin and at stressing the main features related to its bioavailability, absorption, and metabolism, while essential steps determine its absolute health benefits and safety profiles. In addition, despite the scarcity of studies on luteolin bioavailability, the different drug delivery formulations developed to increase its bioavailability are also listed here.

## 1. Introduction

Luteolin is a naturally occurring flavonoid belonging to the class of flavones, often found in combination with glycosides in many fruits, vegetables, and medicinal herbs [1], including carrots, peppers, thyme, broccoli, onion leaves, cabbages, apple skins, rosemary, parsley, and spinach [2]. Regarding botanical families, it is frequently found in Ginkgoaceae, Anacardiaceae, Apiaceae, Asteraceae, Cucurbitaceae, Euphorbiaceae, Fabaceae, Lamiaceae, and others [3].

**Figure 1:**
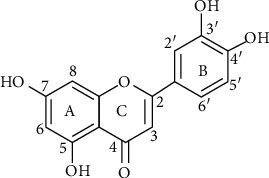
Chemical structure of luteolin.

Structurally, luteolin (molecular formula C_15_H_10_O_6_) has a classic flavone 15C skeleton structure, also known as C6-C3-C6, consisting in two benzene rings and one oxygen-containing ring which presents a C2-C3 double bond [4]. As a tetrahydroxy flavone, luteolin possesses 4 hydroxy groups located at positions 3′, 4′, 5, and 7 (Figure 1), usually found in plants alone as aglycone or bound to one or several sugars as glycoside [5].

Based on huge research studies found in the literature, luteolin has been identified as one of the potential bioactive constituents that potentially contribute to the preventive and pharmacological values of a number of medicinal plant species [6, 7]. As other natural polyphenols, luteolin produces multiple beneficial effects on health [3, 8]. Many preclinical studies report that luteolin exerts a variety of pharmacological actions, including antioxidant and reactive oxygen species (ROS) scavenging [3, 8, 9], antimicrobial [3], anticancer [10–14], cardioprotective [15, 16], neuroprotective [17–19], antidiabetic [3], and anti-inflammatory effects [2, 20, 21]. Recently, an interesting antiviral effect has been proposed against the severe acute respiratory syndrome-coronavirus 2 (SARS-CoV-2) [22]. Hence, it is evident that luteolin can produce numerous health promoting benefits in human beings.

From the other perspective, bioavailability is a crucial step to define the efficacy of any bioactive compound; i.e., bioavailability is the proportion of the substance capable of being absorbed and available in the systemic circulation considering the intestinal endothelium absorption and first-pass metabolism [23]. Various studies have reported that luteolin has low bioavailability, suggesting this as the major limitation of this bioactive compound [24]. This limitation has instigated the need of studying delivery strategies including lipid carriers and nanoformulations for improving the overall efficacy of the luteolin.

To the best of our knowledge luteolin is not considered for the compilation of exhaustive information on the *in vivo* pharmacological activities, features related to its bioavailability, absorption and metabolism, and safety profile. Moreover, comprehension of recent literature on drug delivery formulations developed to increase its bioavailability of luteolin discussed in detail in the review. Taking all the information listed above, this review focuses on the current scientific knowledge and updated comprehensive data available on luteolin to overview the wide therapeutic potential of this natural flavonoid against several chronic diseases based on preclinical experiments, while also collating the data on luteolin bioavailability along with strategies to improve its bioavailability.

## 2. Preclinical Pharmacological Activities of Luteolin: *In Vivo* Evidence

The pleiotropic profile of luteolin has been well established and investigated through various *in vitro*/*in vivo* studies. The multifaceted activity of this flavone is attributed to the interaction with cell protein/pathways whose alteration is often crucial for the development of a specific disease. Even though luteolin is widely known for its antioxidant and anti-inflammatory activities, in the last decade its therapeutic potential as an anticancer flavonoid is growing, with various preclinical studies proving that it exerts proapoptotic and antiproliferative effects, besides being able to inhibit carcinogenesis, metastasis, and angiogenesis [11]. The following sections discuss the various pharmacological actions of luteolin, thanks to its renowned antioxidant, anti-inflammatory, and anticancer effects.

### 2.1. Anti-Inflammatory Activity

Among the luteolin's activity spectrum, anti-inflammatory is one of the most known and investigated by researchers. In a study, Ziyan et al. explored the anti-inflammatory effect of oral administration of luteolin in acute and chronic models of mice. Luteolin, at doses of 10 and 50 mg/kg, suppressed carrageenan-induced paw edema [25]. Further investigations revealed that luteolin selective inhibits cyclooxygenase-2 (COX-2) in inflammatory responses. In addition, due to high cost, lack of access, and side effects related to synthetic anti-inflammatory drugs, researchers belonging to developing countries are still looking for medicinal plant-derived compounds as effective and cheaper alternatives. With this thought in mind, a group of researchers examined luteolin's effect on interleukin-1*β* (IL-1*β*) and tumor necrosis factor-*α* (TNF-*α*) in inflammation induced by lipopolysaccharide (LPS) in male Wistar rats, evidencing a significant reduction of this two inflammatory mediators [26]. Coherently, a plethora of preclinical studies have also focused on the potential application of this flavone in diverse inflammatory diseases. For instance, mastitis is the inflammation of mammary glands triggered by the bacterium *Staphylococcus aureus*, which affect both humans and animals. Guo et al. observed 60 adult postpartum and lactating Bagg albino (BALB)/c mice in order to investigate the effect of luteolin on this particular inflammatory response. Also, in this case, intraperitoneal injection of 25, 50, and 100 mg/kg luteolin injected at 6, 12, 18, and 24 h after the initiation of *S. aureus* infection inhibited the expression of TNF-*α*, IL-1*β*, and IL-6 previously increased by the infection in mammary tissues and mammary epithelial cells [27].

Sepsis is a syndrome of systemic inflammation that might induce organ failures, which is mediated by different pathways in different organs and usually. In an *in vivo* model of sepsis (NALB/c inbred male mice), luteolin was found to inhibit the phagocytosis of macrophages, downregulate the expression of myeloid differentiation factor (MyD)88 and toll-like receptor (TLR)4 in mouse peritoneal macrophages, regulate the expression of cytokines including TNF-*α*, IL-10, IL-1*β*, and IL-6, and reduced the level of peroxisome proliferator-activated receptor (PPAR)-*γ* and signal transducer and activator of transcription (STAT) protein. Therefore, luteolin could counteract sepsis inflammation by inhibiting the PPAR-*γ*/STAT/MyD88 pathway [28].

As regards chronic inflammatory diseases, the effect of luteolin was evaluated also on the activation of pancreatic stellate cells, which is considered to be the initiating stage in the development of chronic pancreatitis, leading to irreversible and progressive pancreatic parenchymal fibrosis. The investigation, performed on female Sprague Dawley rats, showed that luteolin had a protective role in chronic pancreatitis via various mechanisms, including the regulation of inflammatory cytokines release via transforming growth factor- (TGF-) *β*1 signalling pathway [29]. In addition, intestinal mucositis is a toxic consequence of treatments used for cancer, such as chemotherapy or radiotherapy. In this condition, extensive damage occurs in the gut mucosa leading to nausea, bleeding, vomiting, abdominal pain, malnutrition, and sepsis as symptoms. Luteolin was shown to prevent intestinal mucositis induced by irinotecan treatment in female Swiss mice [30] without affecting the effectiveness of the topoisomerase I inhibitor. Therefore, luteolin could be used as an adjuvant in antitumor therapy, to help patients' adherence to treatment.

### 2.2. Anticancer Activity

Although the label cancer includes a wide variety of pathological features, cancer is generally considered a strongly lethal disease. Many chemotherapeutic agents are available on the market, and research is continuously pushing for finding more effective/less toxic therapeutic strategies. Dietary agents are being increasingly investigated for their ability to prevent/manage cancer, since they are safe, less expensive, and easily available [31]. Accordingly, luteolin has been progressively investigated in different types of cancer, and data obtained suggest that it exerts significant anticancer effects. For instance, in an *in vivo* study in male Wistar rats, the protective effect of luteolin was tested against experimental colon carcinogenesis induced by the colon specific carcinogen 1,2-dimethylhydrazine (DMH) [32]. In this model, luteolin was found to protect cell surface and maintain the structural integrity of cell membranes and reduce tumor incidence, thus strengthening the potential protective effect against colon cancer. Besides the anticancer effects against this type of cancer (IC_50_: 5.9 *μ*M), this flavonoid also produced a 24% reduction of colon cancer liver metastasis [33]. Additionally, based on the evidence that aspirin has been associated with a reduced risk of colorectal cancer, other authors investigated the effects of aspirin and luteolin either alone or in combination on male Wistar rats [34]. Findings revealed that coadministration of both biomolecules led to the disappearance of polyps from the colon tissue and normalized cortical convoluted tubules in the kidney tissue. Thus, aspirin seems to be extremely useful when supplemented with luteolin in the treatment of colon cancer, aiming at avoiding gastric and renal toxicity related to the prolonged administration of nonsteroidal anti-inflammatory drugs (NSAIDs). Analogously, luteolin was tested in pancreatic cancer in combination with gemcitabine, a standard-of-care first-line treatment for this type of tumor. The study was performed on male athymic nude mice in an orthotopic model of pancreatic cancer. Results showed a promotion of apoptotic cell death due both to the inhibition of K-*ras*/glycogen synthase kinase- (GSK-) 3*β*/nuclear factor- (NF-) *κ*B signalling pathway and to a reduction of the bcl-2/bax ratio [35]. On the other side, the use of luteolin in combination with 5-flurouracil (5-FU) was also studied in solid Ehrlich carcinoma (SEC) female Swiss albino mice. Results indicated that combined administration of luteolin and 5-FU in SEC model increased levels of p53, p21, caspase-3, damage-regulated autophagy modulator (DRAM), and survivability; on the contrary, tumor volume, weight, thioredoxin reductase one (TR1) activity, and cyclin D1 expression resulted in being reduced with restoration of oxidant/antioxidant indices being also noticed [36].

Some studies have also suggested luteolin as a good complementary therapeutic agent against hepatocellular carcinoma (HCC), a major form of liver cancer highly resistant to chemotherapy [37]. For example, in a study addressing the antioxidant and anti-inflammatory activity of luteolin on N-nitrosodiethylamine induced hepatocellular carcinoma in male Wistar albino rats, luteolin was shown to decrease the number of mast cells, leading to a reduced production of proangiogenic factors [38]. However, since some flavones and flavonols were found to be cytotoxic in several human cell lines, the effect of luteolin as common dietary flavonoid was tested in male Sprague Dawley rats for its effect in HCC. As a result, luteolin led to apoptosis signalling selectively via a mitochondrial-dependent pathway in hepatocytes, confirming its potential in HCC [37].

In another study, the effect of nanoencapsulated luteolin was explored in a xenograft mouse model of head and neck cancer [31]. The xenograft was developed in the flank of athymic nude mice through subcutaneous injections of Tu212 cells. The water-soluble polymer-encapsulated nanoluteolin exerted a significant inhibitory effect on the development of squamous cell carcinoma of head and neck, and this effect was more potent compared to that of hydrophobic luteolin [31].

Ovarian cancer has high incidence and mortality rate among women, and it represents one of the three major malignant tumors in women. To investigate the potential effect of luteolin in this tumor type, the flavone was administered along with other agents in a model of ovarian cancer generated through injection of ES-2 cells to the right hind leg of female nude mice. Histological analysis of tumor tissue indicated that luteolin suppressed proliferation and migration in ovarian cancer cells via downregulating the expression of matrix metalloproteinase 2 (MMP2) and MMP9 [39].

As regards food-related malignancies, experiments performed on C3F mice fed with high- and low-fat content demonstrated that excess energy supply increased the risk of developing mammary tumors [40]. As a matter of fact, insulin-like growth factor-1 (IGF-1) and IGF-1 receptor expression are known to be associated with increasing risk for breast cancer, and both factors are promoted by hyperinsulinemia and insulin resistance. As observed in other cancers, luteolin suppressed tumor formation via its proapoptotic and cell cycle regulatory activity. Similarly, many studies state that red meat and fat are possible dietetic risk factors in the development of prostate cancer by promoting carcinogenesis through the increase of oxidative stress; therefore, administration of antioxidants may be effective in preventing this type of cancer. To validate this hypothesis, in vivo investigation was performed on a heterozygous male transgenic rat for adenocarcinoma of prostate (TRAP). Luteolin was able to suppress early-stage carcinogenesis in prostate cancer and in castration-resistant prostate cancer by inducing apoptosis. Luteolin also induced miR-8080, and this in turn reduced androgen receptor variant 7 (AR-V7) protein resulting in inhibition of tumorigenesis, thus suggesting that both luteolin and miR-8080 might be a new therapeutic strategy for castration-resistant prostate cancer [41]. Among the cancer risk factors, smoking is one of the most dangerous. Since nicotine is a potential inducer of oxidative stress, luteolin's effect in alleviation of disorders induced by nicotine in the liver and lung tissues was investigated in young Wistar albino rats. In line with its antioxidant profile, luteolin exerted an overall beneficial activity, with an enhanced effect in lung tissues [42].

### 2.3. Neuroprotective Effects

Neuroinflammation has demonstrated to exert a key role in the development of several neurodegenerative diseases, such as Alzheimer's disease (AD), Parkinson's disease (PD), stroke, traumatic brain injury, spinal cord injury, demyelinating disorders, and pathologies of the central nervous system (CNS) infections. Among these, traumatic injuries to the spinal cord may cause permanent neurological disabilities. Luteolin has many pharmacological activities, including a memory-improving effect. On this basis, luteolin was combined with palmitoylethanolamide, and their effect was evaluated in CD1 mice, evidencing both neuroprotective and anti-inflammatory properties [43]. In another study performed on the same animal model, co-ultra-micronized palmitoylethanolamide/luteolin demonstrated to ameliorate symptoms of traumatic diseases like spinal cord injury; thus, this composite might be effective in the treatment of spinal cord injury related neuroinflammation [44].

PD and AD are the most common neurodegenerative disorders; even though oxidative stress is considered to have a crucial role, other factors intervene in the development of both pathologies, such as accumulation of misfolded proteins. Coherently, agents that are able to induce autophagy, the process for degradation of intracellular waste material, may be helpful in the elimination of toxic substances from neurons, thus providing a neuroprotective effect. In line with this hypothesis, administration of luteolin with palmitoylethanolamide as endogenous autophagic promoter to male C57/BL6 mice was shown to improve the tissue structure, stimulate autophagy, and ameliorate neurobehavioural functions [45]. As regards AD, other authors investigated the association luteolin and L-theanine on male Sprague Dawley rats to investigate the potential synergistic effect on memory impairment prevention [46]. The rationale of the study is based on the fact that insulin resistance and diabetes are known to be major risk factors for the development of AD, and different studies suggest that both the tested natural compounds could have a role in preventing diabetes-induced neurodegeneration. This hypothesis was tested in male rats that were infused with amyloid-*β* (25-35) to induce AD-like plaques and fed high-fat diets to induce insulin resistance. Combination of luteolin and L-theanine alleviated AD symptoms possibly by improving hippocampal insulin signalling, norepinephrine metabolisms, and decreasing neuroinflammation, thus showing to be a potential therapeutic option.

In another study, the luteolin's effect on neuroinflammation, oxidative stress, synaptic plasticity, and impaired cognitive impairment was tested on adult Sprague Dawley rats and it was determined that luteolin reduced chronic cerebral hypoperfusion-induced microglia overactivation along with other benefits [47]. Moreover, a study conducted by El-Deeb et al. focused on investigating luteolin's effect on autoimmune encephalomyelitis showed that flavone has anti-inflammatory, antiapoptotic, and antineurotrophic activities also in multiple sclerosis [48].

Neuroinflammation is also related to brain injury following intracerebral haemorrhage (ICH), a dramatic event associated with high mortality. An *in vivo* investigation suggested that luteolin engaged tumor necrosis factor receptor-associated factor (TRAF)6 and inhibited its ubiquitination thus downregulating the TRL4/TRAF6/NF-*κ*B and exerting a neuroprotective effect [49]. In cognitive aging and neurodegeneration, a dysregulated overexpression of inflammatory mediators by microglia often occurs. Luteolin is known to be a natural immunomodulatory, and thus, it was thought to be successful in reducing inflammatory microglia. Two different studies performed on young and aging mice (3–6 months old and 22–24 months old, respectively) demonstrated that luteolin inhibited brain microglia activity related to aging and inhibited neuroinflammation, consequently improving cognitive functions [50, 51]. Finally, luteolin was found to provide protection against traumatic brain injury, a major cause of disability which could occur after traumatic experience and could lead to memory impairment and AD, probably thanks to its antioxidant effect [52].

### 2.4. Protection from Metal Overload Toxicity

Luteolin could be also used against negative health effects elicited by toxic heavy metals. For instance, luteolin supplementation was shown to reduce lead overload in hepatic tissues in a male Wistar albino rat by decreasing the release of inflammatory mediators and suppression of apoptotic cascade. Therefore, luteolin could be a dietary supplement that can be used for protection from environmental and occupational lead exposure with no side effects [53]. These results have been confirmed by another investigation carried out on male Wistar albino rats, in which luteolin was shown to protect renal tissues from lead overload through the increase of the antioxidant defense mechanism, the inhibition of the apoptotic pathways and the reduction of inflammatory biomarker levels due to the activation of the nuclear erythroid 2-related factor (Nrf)2/antioxidant response element (ARE) signalling pathway [54].

Lead exposure might also be due to cosmetics, pipes, pigments, and paints and results in adverse reactions on CNS since it replaces zinc and calcium ions enhancing the chances of developing neurodegenerative diseases. Investigations performed to examine luteolin's effect on lead induced neurotoxicity determined that this flavone significantly improves cortical injury following lead intoxication and protected neuronal tissue [55]. In addition to occupational exposure, consumption of cobalt containing foods and drinks, and usage as illegal doping by athletes or racehorses, cobalt is also being used as metal in some orthopaedic prostheses. Unfortunately, ion release from metal implants is associated with neurological alterations such as cognitive decline, incoordination, and depression. *In vivo* administration of CoCl_2_ elicited alterations in cognitive and locomotor activity, with alterations in brain oxidative status and Purkinje cells. Antioxidant substances, such as luteolin and/or gallic acid, are able to provide protection against codependent toxicity through different mechanism such as restoration of Ca^2+^ homeostasis, acetylcholinesterase, and antioxidant enzyme activities, as well as inhibition of lipid peroxidation in the brain [56]. Nickel is used in many industrial applications; however, it is also a potent carcinogenic substance leading to morphological changes in kidney, especially in the glomerulus and the proximal tubules. Prasad et al. focused their attention on investigating whether luteolin can provide protection against nickel chloride-mediated renal oxidative stress. They found that luteolin was capable of preventing biochemical alterations in male Wistar rats by modulating glutathione content and its metabolizing enzymes, scavenging ROS, and reducing tumor promoter marker levels [57].

### 2.5. Protection from Metabolic Dysfunctions

A number of experiments evidenced that luteolin consumption could be a therapeutic approach also for metabolic dysfunctions, such as obesity, diabetes, hepatic steatosis, and postmenopausal metabolic syndrome. Obesity is becoming an important health problem, especially in high-income and developed countries. A comparison among C57BL/6 mice fed low-fat and high-fat diets and also high-fat diet supplemented with 0.002 and 0.01% luteolin for 12 weeks revealed that luteolin ameliorated diet-induced obesity and insulin resistance [58]. Similar results were obtained by other authors using the same models. Notably, inducing the development of brown fat at the expense of the white one might be a beneficial approach in the fight against obesity; luteolin was shown to increase energy expenditure in mice, promote thermogenic program in mouse brown adipose tissues and subcutaneous adipose tissues, and induce white-to-brown fat transition in subcutaneous adipose tissues through an AMP-activated protein kinase (AMPK)/peroxisome proliferator-activated receptor gamma co-activator (PCG)1*α* pathway-mediated mechanism [59]. Moreover, in a study by Zhang et al., dietary luteolin ameliorated insulin resistance in obese C57/BL6 mice via the promotion of AMPK*α*1 signalling also in adipose tissue macrophages, which are regarded as critical in the development of obesity-associated inflammation and insulin resistance [60]. Finally, in C57BL/6 mice subjected to 8 weeks of high-fat diet, both antioxidant and anti-inflammatory activities of luteolin prevented systemic metabolic alterations and vascular dysfunction which is common in obesity [16].

Hepatic steatosis is a pathology commonly observed in obese patients, and it is often associated with insulin resistance. In male C57BL/6 mice fed normal, high-fat, or high-fat plus 0.005% (*w*/*w*) luteolin diet for 16 weeks, luteolin-enriched diet suppressed hepatic lipogenesis and lipid absorption. The flavonoid also prevented adiposity by increasing lipolysis and tricarboxylic acid (TCA) cycle before the formation of lipid droplets in adipose tissue [61]. In analogue investigation, luteolin-enriched artichoke leaf lowered adiposity and dyslipidaemia level by decreasing lipogenesis while increasing fatty acid oxidation, which contributed to ameliorating hepatic steatosis. Luteolin also reduced lipogenesis and increases biliary sterol excretion. Sixteen weeks of supplementation with artichoke leaf extract having high LU content not only prevented obesity and metabolic disorders but also reduced inflammation in high-fat diet-induced obesity animal model, as demonstrated by the reduced plasma interleukin- (IL-) 6, IL-1*β*, and plasminogen activator inhibitor-1 levels [62].

Diabetes mellitus is one of the most widespread chronic metabolic diseases; moreover, many complications are related to this pathology, such as diabetic neuropathy and nephropathy. As a pleiotropic molecule, luteolin was proved to be effective also in this complex pathophysiological disturbance. For instance, oral administration of luteolin reversed glucose intolerance improving insulin sensitivity in an insulin-resistant mouse model [63]. Complications of diabetes-associated neuronal disorders are known to be mediated by apoptosis, and this is known to be mediated in turn by hyperglycemia. Luteolin's effect on neuronal apoptosis was investigated on male Sprague Dawley rats: luteolin administration twice a day for 15 days inhibited hyperglycemia-mediated apoptosis and also improved learning and memory in diabetic neuropathy [64]. Moreover, Li et al. showed that luteolin was successful in improving deficient motor and sensory functions induced by diabetes. This effect might be associated with its Nrf2-dependent antioxidant capacity: luteolin might activate antioxidant defense mechanism to act against the damage caused by reactive radicals, and this activity might contribute to its effect on diabetic neuropathy [65].

Nephropathy is among the most common complications of diabetes mellitus, and it has been observed in 30–40% of diabetic patients. Protective effect of the flavonoid against streptozotocin-induced diabetic renal damage (male ICR mice) was investigated administrating doses of 10 and 20 mg/kg/day for 4 weeks. As a result, luteolin was found to improve insulin signalling pathway and the regulation of receptor-interacting protein (RIP)140/NF-*κ*B [66]. Moreover, luteolin protects the filtration function of the basement membrane and inhibits glomerulosclerosis thus delaying the progression of the pathology [67]. Finally, since diabetes often causes chronic wounds/ulcers, histopathological studies were performed on Wistar albino rats after the administration of ointment containing 0.5% *w*/*w* luteolin, and it was determined that luteolin is potentially considered to have a beneficial effect in enhancing wound healing in diabetes [68] [69] probably due to its antioxidant activity.

Hyperglycaemia-induced vascular disease is a significant cause of morbimortality in diabetes. These disorders are known to be initiated by endothelial dysfunction since the endothelium has a vital role in the regulation of vascular tone and structure. Luteolin is able to protect endothelium-dependent relaxation against high-glucose injury effectively by reducing oxidative stress and increasing the activity of NOS-NO pathway [70]. Moreover, luteolin decreased cardiac apoptosis and inflammation by upregulating antiapoptotic protein fibroblast growth factor receptor (FGFR)2 and leukemia inhibitory factor LIF proteins activating phosphatidylinositol 3-kinase (PI3K)/Akt pathway, being thus able to reduce the infarct size and cardiomyocyte apoptotic rate following ischemia/reperfusion injury in diabetic rats [71].

Metabolic disorders, including insulin resistance, could also occur in menopause due to an enhanced susceptibility to weight gain. Luteolin was shown to reduce adipose tissue inflammation and improved insulin resistance in male mice; however, effects on ovariectomized female mice were not shown. Consequently, luteolin's effect was tested on ovariectomized female C57BL/6 mice. Results showed that dietary luteolin supplementation alleviates adipose tissue inflammation and insulin resistance occurring in mice with loss of ovarian function coupled with a high-fat diet (HFD) intake. This effect may be partly mediated through suppressing macrophage polarization in adipose tissue suggesting that this flavone could be helpful in preventing metabolic syndrome in postmenopausal women [72].

### 2.6. Protection from Cardiovascular and Vascular Alterations

Cardiovascular diseases are actually the first cause of death, and the potent antioxidant effect of luteolin could be of help also in this class of pathologies. Heart failure is among the main cardiovascular syndromes; its increase in frequency in aging population could go from acute to chronic forms. In an experiment, to examine the protective effects of luteolin on chronic heart failure, male Sprague Dawley rats were used, and it was found that long-term administration of this flavonoid might improve cardiac function alteration induced by doxorubicin, also inhibiting apoptotic process in myocardial cells [73]. In fact, doxorubicin is an antracycline antibiotic that is used in cancer treatment, but its clinical efficacy is limited due to its cardiotoxicity. Luteolin's protective effect on doxocardiotoxicity was shown to be mediated by the inhibition of NADPH-dependent lipid peroxidation in a concentration-dependent manner, by the suppression of phlpp1 activity and also the activation of AKT/Bcl-2 signalling pathway [74]. Notably, sarcoplasmic reticulum Ca^2+^-ATPase 2a has an important role in heart failure, and sarcoplasmic reticulum Ca^2+^-ATPase expression was found to be reduced in the failure of myocardium. Luteolin was shown to ameliorate the myocardium fibrosis occurring in a heart failure in vivo model by targeting sarcoplasmic reticulum Ca^2+^-ATPase 2a [75].

On the other hand, oxidative stress (OS) is believed to be one of the main factors responsible for the development of heart failure. ROS increase dramatically during ischemia, subsequently triggering cellular damage during reperfusion. Since antioxidant flavonoids had protective effect against ischemia/reperfusion (I/R) damage, luteolin was investigated in a simulated I/R myocardium model. Results showed that the antioxidant flavone was able to protect cardiomyocytes against I/R-induced injury, and this effect was found to be due to improvement in contractile function of cardiomyocytes, inhibition of oxidative stress, and activation of PI3K/Akt signalling pathway [76]. In another study, luteolin displayed potent cardioprotective effect on myocardial I/R injury, but here, the effect was attributed to downregulation of TLR4-mediated *κ*B/nucleotide-binding oligomerization domain, Leucine-rich Repeat and Pyrin domain containing (NLRP)3 inflammasome [77]. Similarly, luteolin was found to protect against I/R-induced renal injury by blocking ROS generation, inhibiting OS, increasing antioxidant defense, and suppressing inflammation. Accordingly, it also decreased cell apoptosis and endoplasmic reticulum stress [78]. I/R injury seems also to aggravate coronary artery disease, a high mortality and complex pathophysiological process related to Ca^2+^-ATPase activity of the sarcoplasmic reticulum. Also in this case, luteolin is shown to possess cardioprotective effect against I/R injury by inhibiting I/R-induced decrease in sarcoplasmic reticulum Ca^2+^-ATPase activity, and this effect was partially attributed to the PI3K/Akt signalling pathway. It also reduced I/R myocardial infarct size and apoptosis of rat myocardiocytes [79]. Additionally, other investigations suggested that luteolin protected against myocardial I/R injury via promoting the endogenous antioxidant enzyme peroxiredoxin II [80] or by enhancing endothelial nitric oxide synthase- (eNOS-) mediated-S-nitrosylation of Kelch-like ECH-associated protein (Keap) 1 and upregulating Nrf2 and Nrf2-mediated antioxidant signalling pathway [81].

Coronary heart disease is often associated with atherosclerosis, a complex pathological setting, in which dysfunction of lipid metabolism and inflammatory infiltration into arterial walls play a crucial role. Since flavonoids are shown to ameliorate cardiovascular diseases, luteolin's protective effect against the formation of atherosclerosis was tested on 9 week-old male LDLR^−/−^ knockout mice with a C57BL/6 background. As main findings from this study, the authors stated that luteolin was able to provide protection against atherosclerosis by ameliorating the development of atherosclerotic plaque and also protecting aorta from monocyte migration and inflammation via a mechanism that includes AMPK-silent mating type information regulation 2 homolog (SIRT)1 signalling [82]. Usually, atherosclerotic vascular diseases are treated with statins that have lipid regulating activity. However, statins have a number of side effects such as myositis, diabetes, and cataract; therefore, other options are sought for. Luteolin was shown to be a good candidate for this purpose, since it was able to mitigate vascular endothelial injury in Sprague Dawley rats due to its antioxidant effect [83]. Luteolin played an important role also in the inhibition of STAT3 activity, which is thought to play an important role in the development of atherosclerosis [84]. Notably, luteolin demonstrated to increase the elimination of cholesterol, whose accumulation is a key risk factor for atherosclerosis and other cardiovascular diseases [85].

Hypertensive cardiac remodelling is a significant factor for cardiovascular morbimortality; it involves left ventricular hypertrophy and cardiac fibrosis, and OS seems to be implicated in its development. Therefore, luteolin therapeutic potential was evaluated against redox-sensitive pathways in cardiac fibroblasts on an angiotensin II-induced cardiac remodelling rat model. Oral administration of the flavone demonstrated protective effect against cardiac fibrosis and hypertrophy in Angiotensin II-infused rat model probably due to the antioxidant effect [86]. However, also a high-carbohydrate high-fat (HCHF) diet could induce myocardial inflammation finally leading to heart damage. As anti-inflammatory molecule, luteolin could exert its cardioprotective effect against HCHF diet-induced myocardial inflammation by decreasing two of the major proinflammatory cytokines, TNF-*α* and IL-18. In addition, luteolin also decrease lipid peroxidation while significantly increasing endogenous antioxidant biomarkers [87]. Additionally, the antioxidant action of luteolin was thought to be of help also in sepsis-induced cardiomyopathy (SIC), which results from severe sepsis and septic shock and is an invertible type of myocardial depression. In a study performed on male C57BL/6 mice by Wu et al. [88], luteolin administration was found to improve cardiac function, attenuate inflammatory response, alleviate mitochondrial injury, decrease oxidative stress, inhibit cardiac apoptosis, and enhance autophagy. Cardioprotective activity of luteolin might be attributed to its ability to decrease apoptosis and enhance autophagy via the activation of AMP-activated protein kinase signalling, and thus, luteolin can be considered a potential therapeutic agent for treating SIC [88].

Finally, luteolin was considered to be a promising supplement to prevent coronary arterial spasm, since it was shown to antagonize various vasoconstrictors in rat coronary arteries and to augment voltage-gated potassium channels and inward rectifier potassium channels in rat coronary arterial smooth muscle cells [89]. Moreover, luteolin was able to inhibit the development of thrombosis in a FeCl_3_-induced carotid arterial thrombus model [90]. Among the vascular diseases, glaucoma could be also included. Glaucoma is the 2^nd^ most common reason for blindness; this class of pathology encloses the heterogeneous group of disease related to multiple factors such as rise of the vascular dysregulation and intraocular pressure (IOP). Luteolin's antiglaucoma effect was investigated in white New Zealand rabbits, and flavonoid was found to possess ocular hypotensive, neuroprotective, and antioxidant effects via protection of structural integrity and enhancing aqueous outflow. Therefore, luteolin administration can represent a novel approach to attenuate glaucoma [91].

### 2.7. Treatment of Psychiatric and Behavioural Disorders

Depression and anxiety are important disorders that have high incidence throughout the world. Antidepressant drugs are able to relieve some of their symptoms, but they are accompanied by various side effects. In a study focused on investigating its potential antidepressant effect, luteolin was administered to 129Sv/Ev male mice in combination with palmitoylethanolamide. Results showed a significant antidepressant activity at low doses, and thus, this combination might be considered a new approach to ameliorate depression symptoms [92]. In another study, this antidepressant effect was related the potentiation of the gamma-aminobutyric acid (GABA)_A_ receptor-Cl^−^ ion channel complex activity, as possible mechanism responsible from the antidepressant activity of the *Cirsium japonicum* plant itself [93]. Moreover, luteolin was shown to elicit an anxiolytic activity in peripheral inflammation. Peripheral inflammation contributes to anxiety and major depressive disorder, and it is frequently found in individuals who suffer from inflammatory bowel disease. Luteolin was shown to produce analgesic and antidepressant effects in dextran sodium sulfate-induced colitis, and therefore, an open field test was carried out to evaluate the anxiolytic action of luteolin on male mice. According to the findings of this study, due to its anxiolytic action, luteolin can be used in the treatment of peripheral inflammation without producing sedative effects [94]. Notably, this antioxidant showed also antiepileptic properties. In particular, luteolin was shown to reduce seizure frequency and protects peripheral organs from injury [95] and to inhibit the course of kindling, together with a general anticonvulsant effect [96] in pentylentetrazol-induced seizure animal models.

On the other side, this antioxidant could also be of help in drug addiction. For example, methamphetamine (METH) is one of the most widely abused psychostimulant agents that exert many toxic effects, especially on the CNS, and METH-induced neurotoxicity is becoming an important health issue. Since luteolin is known to exert beneficial effects in some neurological disorders, its effects were tested in male Sprague Dawley rats. It was found that luteolin attenuated METH-induced apoptosis and autophagy through the suppression of PI3K/Akt pathway [97]. Continued consumption of addictive drug may also lead to enhancement of behavioural response, a phenomenon called behavioural sensitization. In another investigation performed on METH-sensitized C57BL/6 mice, luteolin was able to attenuate behavioural sensitization-induced by METH via the ERK1/2*Δ*FosB pathway. Moreover, luteolin inhibited the c-Jun N-terminal kinase (JNK) signalling pathway that was found to be involved in METH-induced neurodegeneration [98]. This natural antioxidant was also suggested as a potential sleep promoter to be used against insomnia. In a pentobarbital-induced sleeping mouse model, luteolin was administered orally at 1 and 3 mg/kg, decreased sleep latency, and increased the total sleep time. It was also shown to increase nonrapid eye movement (NREM) sleep time while decreasing wake time. This hypnotic effect was addressed to binding to adenosine A1 and A2A receptors [99]. Finally, since luteolin was shown to protect from behavioural deficits and to enhance the hippocampal neurogenesis in Ts65Dn mouse model of Down syndrome, a therapeutic application of this natural flavone was also hypothesized in this chromosomal disease. These effects were suggested to be related to brain-derived neurotrophic factor (BDNF)/ERK1/2 pathway activation [100].

### 2.8. Hepatoprotective Activity

The liver is the main organ for metabolism and excretion in the body; therefore, it is exposed to environmental toxins and chemotherapeutic agents, whose accumulation may lead to liver diseases and chronic liver damage. Flavonoids such as luteolin combined with membranes in various organs. These flavonoid compounds associate with membranes by hydrogen bonds and save the membrane from deterioration [101]. Similarly natural antioxidants from plant origin (zeaxanthin) were also found to have a protective effect with respect to membrane of cells [102].

The anti-inflammatory and antioxidant activities induced by luteolin were investigated in an *in vivo* model of galactosamine/lipopolysaccharide-induced hepatotoxicity. As expected, luteolin provided a protecting effect against acute liver injury by regulating inflammatory mediators and phase II enzymes [103]. However, the use of luteolin was as a liver-protecting agent as ancient origins, since this flavonoid is abundant in *Achillea millefolium*, a plant traditionally being used to treat liver diseases in India. On this basis, luteolin isolated from the ethanol extract of flowers induced a protective effect against CCl_4_-induced hepatotoxicity probably due to its antioxidant, anti-inflammatory, and immune modulating properties [104]. Luteolin provides a liver defense effect also in association with metformin, which is accounted to provide cancer prevention, antiapoptotic, and anti-inflammatory properties by itself. Accordingly, their combination was found to reduce proinflammatory (IL-1*β*, TNF-*α*, and IL-6) and proapoptotic (caspase-3 and Bax) mediators in hepatic cells [105].

On the other hand, ethanol is one of the most commonly consumed psychoactive substances, with its consumption being linked to hepatic steatosis, which, in turn, could also lead to cirrhosis and hepatocellular carcinoma. Excessive alcohol usage might also contribute to alcoholic liver disease (ALD). In a study performed on six-week-old C57BL/6 mice, it was reported that luteolin may exert beneficial effect on ethanol-induced hepatic steatosis by acting on sterol regulatory element-binding proteins (SREBPs) that control the biosynthesis of cholesterol, fatty acids, and triglycerides, as it was found to ameliorate ethanol-induced hepatic steatosis and injury; therefore, it might be helpful in ALD intervention [106]. In a similar way, nonalcoholic steatohepatitis (NASH) is a liver inflammation and damage caused by an accumulation of fat and belongs to the class of nonalcoholic fatty liver diseases. NASH is strongly related to metabolic syndrome such as obesity, insulin resistance, type 2 diabetes, and dyslipidaemia, but this disorder might also lead to the development of hepatocellular carcinoma. In an *in vivo* study, luteolin targeted proinflammatory IL-1 and IL-18 pathways along with its antioxidant effect and protected the liver against NASH induced by a high-fat diet [107]. Notably, connexin (Cx) 32 is a hepatocyte gap-junction protein suggested to prevent hepatocarcinogenesis. On this basis, some experiments were carried out to clarify the role of Cx32 and the chemopreventive effect of luteolin on the progression of NASH and NASH-related hepatocarcinogenesis. Results confirmed a liver-protecting activity for luteolin; moreover, Cx32 downregulation was detected during steatohepatitis in Wistar rats, indicating that Cx32 plays a protective role against NASH progression [108]. Coherently, multifaceted activity of luteolin was evaluated also in hepatocellular carcinoma (HCC), the most common type of primary liver cancer that occurs frequently in people with chronic liver diseases, such as cirrhosis caused by hepatitis B or hepatitis C infection and linked to an important fatality rate. *N*-Nitrosodiethylamine (DEN) is a potent hepatocarcinogen and is known to be present in tobacco smoke, water, cheddar cheese, fried meals, and some alcoholic beverages. Tested DEN-induced hepatocellular carcinoma, luteolin was found to stabilize and restore antioxidant defense system and reduced DEN-induced increased ROS generation which occurred during hepatocarcinogenesis [109]. Finally, this polyphenol provided a hepatoprotective effect against hepatotoxicity induced by methamphetamine (METH), an often used illicit drug, by modulating oxidative phosphorylation, cytochrome P450, and certain signalling pathways [110].

### 2.9. Protection from Musculoskeletal Diseases

Osteoporosis is a complex systemic skeletal disease characterized by reduced bone mass, disruption in bone microarchitecture, and increase in bone fragility. Some flavonoids are known to prevent bone loss in ovariectomized animals, and in addition, they also inhibit osteoclast differentiation. In a study, the oral administration of luteolin led to a significant increase in bone mineral density, while also prevented ovariectomized-induced increase in bone turnover [111]. Therefore, this flavonoid might be used as an alternative to current therapeutic agents in the management of postmenopausal bone loss.

Some investigation explored the effect of luteolin also in muscular system disorders. For instance, luteolin's effect on rat bladder smooth muscle contractility was investigated in male Sprague Dawley rats, showing to inhibit isolated detrusor muscle contraction induced by Ach and KCl [112]. In another study, performed on male Hartley guinea pigs, focused on evaluating whether luteolin could have an effect on gallbladder motility, the flavone relaxed cholecystokinin- or KCl-induced tension by blocking extracellular Ca^2+^ entry and also by blocking intracellular Ca^2+^ release. Authors concluded that, since it possesses oestrogen-agonist activity, supplementation of luteolin could affect gallbladder contractility, whose dysfunction leads to gallstones [113]. Finally, protective activity of a series of different compounds, luteolin included, was explored on a glucocorticoid-induced muscle atrophy model. Skeletal muscle atrophy usually occurs after starvation, aging, immobilization, and denervation and consists in the loss of muscle mass. Luteolin was able to attenuate skeletal muscle atrophy induced by dexamethasone and exerted this kind of protection via antioxidant and antiapoptotic mechanisms [114].

### 2.10. Organ Transplantation

Given the activity of luteolin in cardiovascular context, potential flavone application has been also evaluated in heart transplantation. Transplantation of the heart is performed for end-stage cardiac failure, but hypothermic preservation of the heart is limited to 4-6 h, and cell death usually occurs due to Ca^2+^ accumulation in time. Luteolin demonstrated to attenuate calcium overload over a preservation period of 6 h and suppress accumulation of regulatory proteins and specific enzymes required for cardiomyocyte calcium circulation. Therefore, the addition of luteolin to preserve the heart may extend the time of preservation [115]. In another study on the same topic performed with specific pathogen-free grade Sprague Dawley rats of both sexes, luteolin was found to improve long term-heart preservation (≤18 h) probably due to inhibition of hypoxia-dependent L-type Ca^2+^ channels [116].

Beneficial effects were found also in transplantation of the kidney. The success of renal transplantation might be seriously compromised by inflammatory responses and damage to tissues in the long term; I/R injury and increased ROS level are among the major causes that lead to this complication. Thus, given the potent antioxidant and anti-inflammatory activity of luteolin, its administration in animal models resulted in reduction in TNF-*α*, IL-1*β*, and IL-6, whose level dramatically increased in I/R injury, and restored cellular viability of the damaged renal tissue [117].

### 2.11. Miscellaneous Studies

A great number of *in vivo* studies are mainly concentrated in cancer, neurodegeneration, and metabolic and cardiovascular diseases; however, several spare studies have also focused to explore luteolin therapeutic potential in other pathologies, often linked to inflammation. For instance, asthma is a long-term inflammatory disease which affects people of all ages and often starts during childhood. Overproduction of airway mucus is a common symptom associated with asthmatic mortalities and morbidities that cannot be prevented completely. Mucus normally plays an important role in the prevention of pathogenic microorganism invasion; however, chronic mucus hypersecretion increases the risk of developing lung cancer. In a study on an adult BALB/c mouse asthma model [118], luteolin markedly inhibited the excessive production of mucus by partially inhibiting gamma-aminobutyric acid-A receptor (GABA_A_R) activities and thus may be used in asthma patients for the management of mucus overproduction. However, asthma could also have an allergic trigger. Therefore, antiallergic effect of luteolin was examined in mice and it was found that its administration reduced infiltration of inflammatory cells and decreased Th2 cytokines, thus exerting an antiallergic effect [119]. However, luteolin could also elicit a general improvement in lung function similar to those provided by bronchodilators [120].

On the other hand, some research also evidenced luteolin activity in nephrology. Renal anaemia is a complication of chronic kidney disease and decreased life expectancy and life quality of the patients. Erythropoietin deficiency is considered a major cause for its occurrence, since erythropoietin regulated red blood cell production. In an experiment performed on male Kunming mice, it was investigated whether dietary luteolin could alleviate renal anaemia induced by chronic HgCl_2_ exposure. In line with its pharmacological profile, luteolin increased the expressions and strengthened the activity of antioxidant enzymes, attenuated oxidative stress by activating HIF-2*α* gene, and ameliorated renal anaemia [121]. Notably, a nephroprotective effect in acute renal failure induced by gentamicin was also found [122].

Electromagnetic field (EMF) radiation has an oxidative effect and has a deleterious effect on the body by inducing oxidative damage to carbohydrates, proteins, lipids, and enzymes and results in cellular function impairment and cell death. In a study performed to evaluate EMF's effect on rat cerebellum and antioxidant effect of luteolin against damage, Global System for Mobile Communications (GSM) standard operating in 900 MHz frequency was tested on male Wistar albino rats [123]. At the end of the experiment, it was determined that 900 MHz EMF radiation had hazardous effects on rat cerebella and luteolin administration for 28 days displayed a neuroprotective effect against cerebellar damages induced by EMF [123].

Cutaneous leishmaniasis is a common skin infection in both humans and animal caused by the *Leishmania* parasite, and it has been estimated that 1.5-2 million people get infected and 60.000 of them die every year. An *in vivo* study showed that luteolin could be effective as meglumine antidote and cryotherapy, which are considered to be classical treatment methods in this disease. In addition, this flavone can also be used in combination with these two classical treatment options to yield better results [124]. Beneficial effects induced by luteolin were also in a neuropathic pain model, where luteolin administration was found to reduce neuropathic pain, also potentiating the effects of morphine [125].

## 3. Bioavailability of Luteolin

As extensively described, luteolin is able to produce multiple beneficial effects on health. But beyond this extensive knowledge on therapeutic potentialities of luteolin, the issues related to its bioavailability, absorption, and metabolism are a key to determine their absolute health benefits and safety profiles [126]. Despite the importance they could have, studies on the bioavailability of luteolin in human and animal models are very limited and this area remains to be unfocused. Briefly, the bioavailability of dietary phytochemicals can be defined as the proportion of the substance capable of being absorbed and available in the systemic circulation considering the intestinal endothelium absorption and first-pass metabolism. Absorption involves entry of a molecule across the biological membrane barrier, which, in case of dietary compounds, is the gut epithelium. Thus, after absorption, the molecule needs to pass the gut wall and to overtake hepatic metabolism, defined as the first-pass effect. On this basis, it becomes clear that, to understand the bioavailability of luteolin, a deeper focus is necessary on understanding its absorption and metabolism.

### 3.1. Absorption of Luteolin

**Figure 2:**
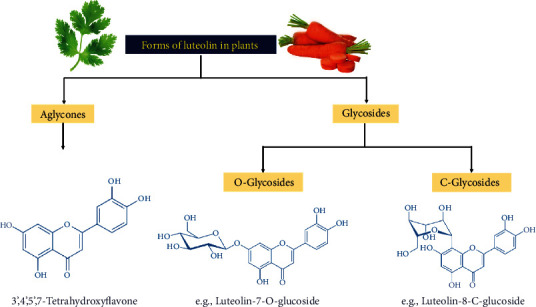
General classification of the typical forms of luteolin.

Absorption of polyphenols across the gut epithelium is the first biological barrier for oral administration; therefore, understanding the intestinal uptake and efflux mechanisms is crucial to determine the efficacy of these beneficial compounds [127]. Luteolin widely occurs in vegetables, fruits, and natural herbs either in a free-aglycone form or in glycoside structures represented by one or more conjugated sugars. As represented in Figure 2, the typical forms of luteolin glycosides mainly occur by glycosylation of aglycone (1) through free hydroxyl (OH) groups, named as O-glycosides, and/or (2) through C-C bond termed as C-glycosides [3]. These glycosylated forms of luteolin are commonly found in the leaves of celery, green pepper, parsley, thyme, peppermint, and perilla [128].

Shimoi et al. investigated the intestinal absorption of luteolin and luteolin 7-*O*-*β*-glucoside in rats and in two human volunteers [128]. In this study, luteolin and *O*-methyl luteolin (chrysoeriol) isolated from perilla seed were used to evaluate intestinal absorption and metabolite profiles. The intestinal absorption of luteolin and luteolin 7-*O*-*β*-glucoside from the mucosa to the serosal side was investigated by using rat everted small intestine incubated in a specific solution containing 1 mM luteolin or luteolin 7-O-*β*-glucoside. High-performance liquid chromatography (HPLC) was used to measure the constituents appearing in the serosal side; chromatograms showed the presence of the luteolin's peak together with two metabolite peaks, detected at different time points. Here upon, the two metabolites were demonstrated to be glucuro- and/or sulpho-conjugates of luteolin since the treatment with *β*-glucuronidase and sulfatase led to the disappearance of metabolite peaks while luteolin peak increased. With regard to luteolin 7-*O*-*β*-glucoside absorption, luteolin 7-*O*-*β*-glucoside peak and the two same metabolite peaks were detected by HPLC; again, luteolin peak was increased while metabolites disappeared following *β*-glucosidase treatment. These results suggest that luteolin is absorbed after luteolin 7-*O*-*β*-glucoside hydrolyzation to luteolin and also that luteolin glucuronide metabolites occurred during absorption [128]. Additionally, in the same study, blood samples were collected from rats 3 h after administration of 50 *μ*mol/kg of luteolin or luteolin 7-*O*-*β*-glucoside by gastric intubation and from humans 3 h after ingestion of 50 mg of luteolin in starch solution. According to HPLC elution profiles of rat plasma, free luteolin and one luteolin conjugate peaks were detected. After treatment with *β*-glucuronidase/sulfatase, peaks of luteolin and methylated conjugate (chrysoeriol) were markedly raised but not luteolin 7-O-*β*-glucoside peak was detected. Furthermore, HPLC chromatogram and mass spectra analysis showed that free luteolin and luteolin monoglucuronide were found in both human serum and rat plasma [128].

In a crossover study, two different extracts of artichoke leaf (ALE) containing luteolin-7-*O*-glucoside and caffeoylquinic acids were prepared separately to supply 14.4 mg (ALE A) and 35.2 mg (ALE B) luteolin equivalents [129]. ALE A and B were administered randomly to 14 healthy volunteers, and then, blood and urine samples were collected for 24 h. HPLC analysis of human plasma and urine samples after the ingestion of extracts A and B showed that luteolin was only obtained as phase II metabolites (sulfates or glucuronides) and free luteolin was only detected after *β*-glucuronidase treatment by enzymatic hydrolysis of metabolites [129]. The time courses of total luteolin concentrations in plasma for each extract showed parallel lines, and also, the highest plasma concentration was reached at 59.08 and 156.58 ng/mL for luteolin, respectively, within 30–40 min. Moreover, according to pharmacokinetic parameters of luteolin, the elimination half-life (*t*_1/2_) was quantified as 2–3 h for both treatments, and the recovery percentages of luteolin-7-*O*-glucoside were calculated as 1.727% and 1.997%, respectively, for extracts A and B within 24 h in urine. Consequently, besides the rapid absorption feature of luteolin in humans, its graph of elimination showed a biphasic distribution profile and a slow elimination phase [129].

*In situ* single-pass intestinal perfusion (SPIP) technique was performed on a selected intestinal segment of rats to elucidate whether luteolin absorption occurs at a specific site of the intestine among the duodenum, ileum, colon, or jejunum [130]. The parameters for effective permeability (*P*_eff_) and the intestinal absorption rate constant (*k*_a_) of luteolin were significantly higher and more efficient in the duodenum and jejunum segments than those in the colon and ileum. Specifically, in the jejunum segment, *P*_eff_ and *k*_a_ values did not change by the increased luteolin concentrations (2.5, 5, and 10 *μ*g/mL). The addition of an ATP inhibitor to the perfusion buffer displayed the passive absorption mechanism of luteolin in the rat intestine. Moreover, the study is aimed also at investigating the difference in absorption of luteolin when it is administered as peanut hull extract (PHE) form and as pure luteolin. Bioavailability of luteolin from PHE was found to be significantly higher than the pure luteolin by pharmacokinetic analysis after a single dose of oral administration of each one to rats [130]. The peak plasma concentrations (*C*_max_) and the area under the concentration curve (AUC) of luteolin from PHE were quantified as significantly and considerably higher (8.34 ± 0.98 *μ*g/mL and 20.3 ± 1.3 *μ*g/mL/h, respectively) than that of pure luteolin (1.97 ± 0.15 *μ*g/mL and 10.7 ± 2.2 *μ*g/mL/h, respectively) [130].

An *in vitro* study investigated the effect of *Artemisia afra* extract on the bioavailability of luteolin by comparing the extract with pure luteolin and its derivatives in human intestinal epithelial Caco-2 cells [131]. Pure luteolin aglycone, luteolin-7-*O*-glucoside, and *A. afra* un-hydrolyzed and acid-hydrolyzed aqueous extract forms (with equivalent concentration of luteolin) were added to the donor side of inserts and blank medium into the basolateral side [131]. As a result of HPLC and LC-MS analyses of each sample at 3 different concentrations, uptake of luteolin and its 7-*O*-glucoside in the extracts were significantly faster and quantitatively higher by 1.6- to 2-fold for the apical to basolateral permeability (*P*_app_) coefficients than that of the nonplant solutions [131]. The parent compounds in all samples were metabolized more than 90% after 2 h incubation of Caco-2 cells, and their metabolite profiles were nearly similar. However, unchanged luteolin and luteolin-7-*O*-glucoside were also detected on the basolateral sides of cells exposed to the pure luteolin/glycoside solutions indicating that; as regards glucuronidation, luteolin metabolism in the Caco-2 cells was more efficient in *A. afra* aqueous extracts [131].

Although the chemical structures of luteolin and apigenin are very similar, and *Flos Chrysanthemi* extract (FCE) contains nearly the equal concentrations of them; their absorption and metabolism profiles showed remarkable differences in rats after oral administration of the extract [132]. For this aim, a SPIP rat model on the selected jejunum segment was set up and it was perfused with Krebs-Ringer's buffer containing 20 *μ*M luteolin or 20 *μ*M apigenin; then samples were collected at 15 min intervals for 1 h. According to HPCL analysis, permeability of luteolin showed a *k*_a_ value of 7.96 × 10^−2^/min and a *P*_eff_ value of 4.87 × 10^−3^ cm/min which resulted to be about half of the apigenin values. These findings were confirmed by analysis of the oral bioavailability of luteolin, which showed significantly lower luteolin/apigenin ratio of AUC/dose as 0.0889 (oral administration of FCE) and 0.141 (intravenous injection) as well as lower mean residence time (3.43 h) and *t*_1/2_ (2.39 h) compared to apigenin (7.31 and 5.73 h, respectively) [132].

Intestinal absorption of luteolin derived from *Aloysia triphylla* infusion was investigated in both healthy and dextran sodium sulphate- (DSS-) induced colitis rats by using the SPIP model [133]. Intestinal absorption of luteolin 7-diglucuronide was not found to significantly differ between the healthy and colitis rats. The percentages of luteolin 7-diglucuronide absorption after perfusion of *A. triphylla* infusion through the intestinal lumen were quantified as 5.57% in healthy rats, 6.15% in perfused with DSS+*A. triphylla* infusion solution, and 4.48% in rats perfused with *A. triphylla* infusion after 7-day treatment with DSS. After rats were received *ad libitum A. triphylla* infusion (including 217 *μ*M of luteolin) for 14 days, only luteolin aglycones (as 279 nmol/24 h) and diosmetin were excreted in the feces. In urine samples, native *A. triphylla* flavone diglucuronides or luteolin was not detected, whereas peaks for various glucurono- and/or sulpho-conjugated metabolites were recorded by HPLC [133]. After enzymatic hydrolysis, released flavone aglycones such as luteolin were identified from both native *A. triphylla* diglucuronides and conjugated metabolites in effluent (deriving from intestinal perfusion) and urine samples. These results revealed that luteolin diglycosides are hydrolyzed, while aglycones are conjugated in the intestinal phase. Furthermore, luteolin showed low urinary excretion, while glycosylation seems to affect the fecal excretion of luteolin [133].

Another study demonstrated that luteolin and its glycosides are rapidly absorbed in rats after oral administration of *Chrysanthemum morifolium* flower extract equivalent to 22.8 and 58.3 *μ*mol/kg bw of luteolin and luteolin-7-O-glucoside, respectively [134]. Luteolin and luteolin-7-*O*-glucoside reached their max concentrations in the animal plasma samples (0.76 ± 0.27 *μ*M and 9.88 ± 1.35 *μ*M, respectively) after 1 h of treatment. Furthermore, metabolites containing luteolin monoglucuronide, luteolin glucoside glucuronide, luteolin diglucuronide, luteolin glucoside sulfate, and luteolin glucuronide sulfate were also detected in rat plasma samples 2 h after oral administration. Moreover, the concentrations of luteolin and luteolin-7-*O*-glucoside were found to be significantly decreased in the apical side of Caco-2 cells, while they increased in the basolateral side in a time-dependent manner. By the way, the aforementioned conjugates also appeared in the basolateral side of Caco-2 cells, confirming the results of *in vivo* experiments and suggesting that these conjugates may circulate also in the human system [134]. However, investigations on *in situ* mice perfusion model showed that the intestinal absorption after treatment with 10 and 20 *μ*M of pure luteolin presented a significant difference between the small intestine and colon segments of mice [135]. The amounts of luteolin absorbed increased dose-dependently in both small intestine and colon; however, both the amount and percentage of luteolin absorbed were significantly higher in the small intestine compared to the colon. Additionally, the intestinal first-pass metabolism of luteolin and the process of luteolin glucuronidation were investigated and three new luteolin glucuronides (3′,7- and 4′,7-diG's and luteolin-5-O-monoglucuronide) were identified by using expressed human UGTs [135].

In another *in vivo* study, luteolin was orally administered at the dose of 200 mg/kg to rats, and 12 h later, bile, urine, and fecal samples were collected [136]. Five metabolites, luteolin-7-*O*-*β*-D-glucuronide, luteolin-4′-*O*-*β*-D-glucuronide, luteolin-3′-*O*-*β*-D-glucuronide, 3′-*O*-methyl-luteolin-7-*O*-*β*-D-glucuronide, and *O*-methyl-luteolin-3′-*O*-*β*-D-glucuronide were isolated from urine, while 4′-*O*-methyl-luteolin-7-*O*-*β*-D-glucuronide was isolated from bile. In addition to glucuronide metabolites, chrysoeriol (3′-*O*-methyl-luteolin) and diosmetin (4′-*O*-methyl-luteolin) were also identified by nuclear magnetic resonance (NMR) and HPLC-UV analysis of the samples [136]. Accordingly, the 8 metabolites mentioned were also detected in plasma samples of rats following either intravenous or oral administration of luteolin; in particular, luteolin-3′-*O*-*β*-D-glucuronide was identified as the most abundant metabolite in systemic circulation and in most tissues. Systematic pharmacokinetic examinations showed that luteolin was rapidly and efficiently absorbed from a rat intestine and then extensively metabolized via glucuronidation or methylation. Therefore, the bioavailability of free luteolin, detected around the 17.5%, suggested a large-scale first-pass metabolism of this compound in the intestine and/or liver during absorption [136].

### 3.2. Metabolism of Luteolin

**Figure 3:**
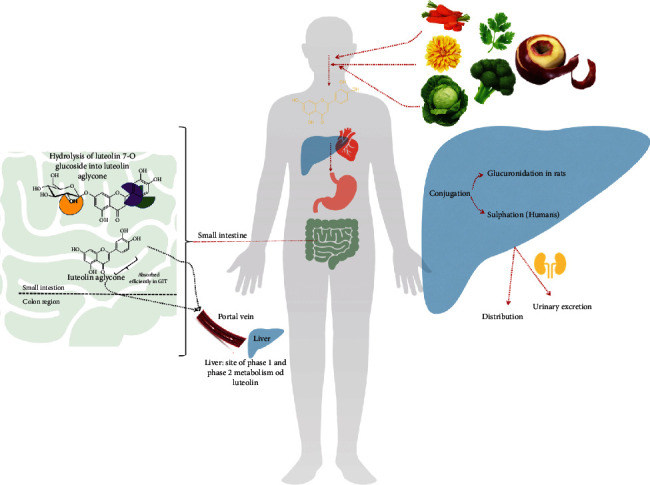
Simple overview of flavonoid postabsorptive metabolism.

After absorption, cytochrome P450 monooxygenases in the liver can extensively metabolize flavonoids by phase I metabolism. It is known that 18 families with many different isoforms of cytochrome genes are involved in flavonoid metabolism [137, 138]. However, products of phase I metabolism-derived oxidation tend to be minor metabolites since it is thought that rapid glucuronidation, sulphation, or methylation of potential phase I substrates by phase II conjugating enzymes, including urine-5′-diphosphate glucuronosyltransferases (UGTs), sulphotransferases, and catechol-O-methyltransferases (COMTs) could represent the major metabolism routes [139, 140]. Mainly, glucuronide conjugates are present abundantly in the plasma and urine [141]. Studies on the metabolism of luteolin are limited; thus, knowledge in the filed remains uncertain. A brief visualization of this flavonoid metabolism is shown in Figure 3.

In the study published by Hayasaka et al., three different luteolin glucosides and luteolin aglycone were isolated from green pepper leaves and administered to rats; in addition, luteolin aglycone was also administered to humans [142]. As main findings, it was found that luteolin aglycone or glucosides were mainly metabolized to luteolin glucuronide in rats, whereas luteolin-3′-O-sulfate was found to be abundant in human plasma after luteolin aglycone administration. Besides, it was suggested that luteolin glucuronide and luteolin sulfate remain for a long period of time in both the human and rat body.

The metabolic pathway of luteolin following oral administration of luteolin aglycone or luteolin-7-*O*-glucoside to rats was also investigated [143]. Luteolin-3′-*O*-glucuronide was found to be the main metabolite in the plasma, but luteolin-4′-*O*-glucuronide and luteolin-7-*O*-glucuronide were also detected. Moreover, glucuronides' effects on gene expression levels were investigated and it was found that luteolin-7-*O*-glucuronide reduced the expression levels of proinflammatory mediators, such as IL-6, IL-1*β*, NF-*κ*B1, Ccl2, Ccl3, Ccl5, and JunB more than other luteolin glucuronides, but less than luteolin itself.

The role of glucuronidation and methylation pathways of luteolin in rats were also investigated, and two possible pathways were suggested [144]. In the first one, luteolin is glucuronidated by the action of UGTs to luteolin-7-glucuronide, luteolin-4′-glucuronide, and luteolin-3′-glucuronide, the latter found as the most abundant in the rat plasma and bile. Then, luteolin-7-glucuronide is methylated to chrysoeriol-7-glucuronide and diosmetin-7-glucuronide by the action of COMTs. The second potential pathway foresees an initial methylation of luteolin to chrysoeriol and diosmetin by the action of COMTs and then the glucuronidation to the respective chrysoeriol and diosmetin glucuronides by the action of UGTs. Thus, both glucuronidation and methylation of luteolin were balancing each other with conversion of metabolites to one another by the actions of COMTs and UGTs, with glucuronidation being the dominant pathway. Finally, luteolin was found to be metabolized by CYP1 enzymes in human breast adenocarcinoma cell lines MCF-7, MDA-MB-468, and breast epithelial cell line MCF10A [145]. In particular, it was reported that luteolin could be metabolized into 6 hydroxyluteolin by recombinant CYP1B1, but not by CYP1A1 and CYP1A2, and that hydroxylation of luteolin at the 6 position of the A ring is crucial for the antiproliferative activity in breast cancer cells. In addition, CYP1-inhibitors *α*-napthoflavone and/or acacetin reduced the antiproliferative activity of luteolin. Molecular modeling showed CYP1B1 favored a ring orientation to the heme group compared to CYP1A1; therefore, activation of CYP1B1 and conversion of luteolin to the hydroxylated form may enhance antiproliferative activity against breast cancer.

### 3.3. Strategies to Enhance Luteolin Bioavailability

**Figure 4:**
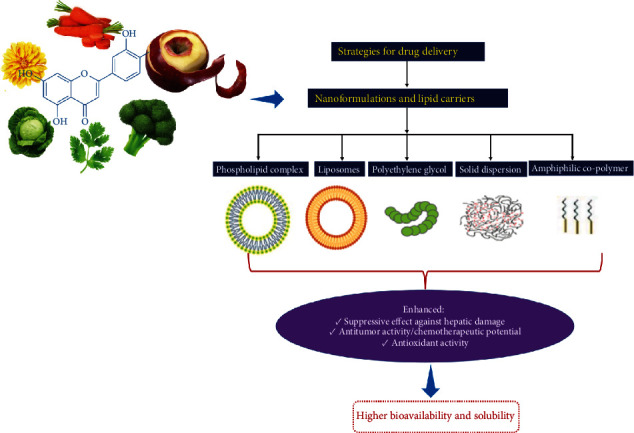
Proposed strategies to increase luteolin's delivery.

Low bioavailability is one of the major limitations in the therapeutic use of luteolin due to extensive first-pass metabolism by phase II enzymes [146]. To overcome this issue, different delivery strategies have been developed and investigated, including lipid carriers and nanoformulations (Figure 4).

Of the studies performed so far, a group of authors assessed the bioavailability of nanostructured lipid carrier (solid) and microemulsion (liquid) formulation of luteolin in rats [147]. The oral bioavailability of luteolin was increased in both forms, but microemulsion reached better results. Besides, solubilized drug concentration was found to be higher for microemulsion which suggests fast absorption and high bioavailability. Also, the complexation of luteolin with phospholipid matrix (LPC) enhanced solubility, bioavailability, and efficacy of luteolin. For instance, LPC treatment resulted in a higher decrease in ear and paw oedema in rats compared to that of treated with pure luteolin [148]. Later, the same research group demonstrated that oral treatment of albino rats with LPC decreased hepatic damage more effectively than luteolin-treated rats by targeting inflammation sites of the liver [149]. Likewise, liposome encapsulation has been reported to have significant antitumor activity against colorectal carcinoma cells (CT26) compared to free luteolin [150]. Luteolin was also coated with amphiphilic copolymer poly(ethylene oxide) monomethyl ether-poly(lactide-co-glycolide) (MPEG-PLGA) to prepare luteolin micelles with improved dispersion in water and sustained drug release [151]. As a result, drug concentration and cell uptake at the injury site in cerebral ischemia-reperfusion (IR) injury were enhanced when compared to free luteolin in pheochromocytoma cells. In another study, polyethylene glycol (PEG) capsulated luteolin was tested in immortalized human keratinocytes, and higher caspase-3/caspase-14 induction in apoptosis was reported, suggesting an enhanced chemotherapeutic potential [152]. Recently, luteolin solid dispersion (LT-SD) was prepared with polyethylene glycol 4000 at various concentrations and methods [153]. It was reported that LT-SD prepared with microwave radiation technique showed higher dissolution. Overall, all LT-SDs showed higher antioxidant activity compared to pure luteolin. As evidenced from the above-mentioned studies, different formulation strategies may enhance the efficacy of luteolin probably by increasing its solubility at the target sites and consequently its bioavailability.

## 4. Conclusion

Findings cited in this review advise that luteolin plays various health-promoting effects. These beneficial roles include protective action against cancer, oxidative stress, metal overload toxicity, behavioural disorders, neuroinflammation, inflammation, and cardiovascular diseases. Luteolin also plays important roles in preventing metabolic disorders including obesity, diabetes, and hepatic steatosis; besides to be also well-established from the findings discusses here, the mechanism of action of luteolin varies in diseased and normal cells, where it becomes cytotoxic to cancer cells but safer to normal ones. Taking this in mind, noteworthy is the issue related to luteolin bioavailability, a crucial step to define its effective biological properties and efficacy. Absorption and metabolic studies showed that luteolin has very limited bioavailability. Hence, a number of drug delivery strategies have been employed to improve the bioavailability of the luteolin in the human system. It is evident from data discussed here that nanoformulations and lipid carriers, such as liposomes, have been highly addressed to improve luteolin bioavailability. However, despite the numerous studies conducted to deeper knowledge on luteolin' pharmacological potential, the exact mechanism to treat various pathologies is still unclear. Further, clinical trials are still very limited to establish an effective dose of luteolin to be given for the treatment of certain disorders. Further studies are needed to answer to open questions and to establish an effective dose of luteolin to be administered for controlling certain disorders.

## Data Availability

The data used to support the findings of this study are available from the corresponding author upon request.
